# Kea cooperate better with sharing affiliates

**DOI:** 10.1007/s10071-016-1017-y

**Published:** 2016-07-29

**Authors:** Raoul Schwing, Elodie Jocteur, Amelia Wein, Ronald Noë, Jorg J. M. Massen

**Affiliations:** 1 Comparative Cognition, Messerli Research Institute, University of Veterinary Medicine, Medical University of Vienna, University of Vienna, Vienna, Austria; 2 Haidlhof Research Station, University of Veterinary Medicine, University of Vienna, Bad Vöslau, Austria; 3 Département Ecologie, Physiologie et Ethologie, IPHC, Strasbourg, France; 4 Faculté Psychologie, Université de Strasbourg, Strasbourg, France; 5 Department of Cognitive Biology, University of Vienna, Vienna, Austria

**Keywords:** Cooperation, Reward division, Loose-string paradigm, Kea, Parrot, Affiliation

## Abstract

Controlled studies that focus on intraspecific cooperation tasks have revealed striking similarities, but also differences, in abilities across taxa as diverse as primates, fish, and birds. Such comparisons may provide insight into the specific socio-ecological selection pressures that led to the evolution of cooperation. Unfortunately, however, compared to primates data on birds remain relatively scarce. We tested a New Zealand psittaciform, the kea, in a dyadic cooperation task using the loose-string design. During trials our subjects were in separate compartments, but obtained a common reward that could be divided multiple ways, allowing the examination of reward division effects. Ten individuals were tested twice in 44 combinations of partners. Dyads with a high affiliation score attempted to cooperate more often and were also more often successful in doing so. Furthermore, dyads that shared rewards more equally seemed to be more likely to attempt cooperation in the next trial. Like other bird and some monkey species, but unlike, for example, chimpanzees, kea did not spontaneously show understanding of either the role of the partner or the mechanism behind the cooperation task. This may point to true disparities between species, but may also be due to differences in task design and/or the amount of exposure to similar tasks and individual skills of the subjects.

**Electronic supplementary material:**

The online version of this article (doi:10.1007/s10071-016-1017-y) contains supplementary material, which is available to authorized users.

## Introduction

The cognitive mechanisms used during cooperation by non-human vertebrates have long interested researchers (for reviews, see Noë 2006; Brosnan and Bshary 2010; Schino and Aureli 2010; Cronin and Sánchez 2012; McNally et al. 2012; Seyfarth and Cheney 2015). Intraspecific cooperation has gained a lot of attention in the past decades from experiments on several distantly related families, such as Hominidae (great apes; Chalmeau et al. 1997a; Melis et al. 2006a, b; Hare et al. 2007), Callitrichidae (tamarins and marmosets; Chalmeau et al. 1997b; Mendres and de Waal 2000; Cronin et al. 2005; Cronin and Snowdon 2008), Hyaenidae (hyenas; Drea and Carter 2009), Canidae (dogs; Ostojić and Clayton 2014), Elephantidae (elephants; Plotnik et al. 2011), Delphinidae (dolphins; Kuczaj et al. 2014), Corvidae (rooks and ravens; Seed et al. 2008; Scheid and Noë 2010; Massen et al. 2015; Asakawa-Haas et al. 2016), and Seranidae (sea bass and groupers; Vail et al. 2014). Such controlled studies that focus on intraspecific cooperation tasks have revealed strikingly similar cooperative abilities across vertebrate taxa. However, there remain disagreements as to the underlying mechanisms and motivations that make successful cooperation possible (De Waal and Davis 2003; Noë 2006; Emery et al. 2007) as success often requires specific training (Crawford 1937; Chalmeau et al. 1997b; Melis et al. 2006b). Nonetheless, understanding the mechanism of the task, i.e. the underlying cause and effect, or the need for the partner is not always required for cooperation to be successful (Chalmeau et al. 1997b; Visalberghi et al. 2000; Noë 2006). However, characteristics of individual temperament can affect the success, or lack thereof, of a cooperative interaction (Hare et al. 2007; Scheid and Noë 2010). In the light of this, unresolved issues may be clarified by revising methodologies in a way that is directly comparable to previous work while focussing on a narrower range of factors (cf. Asakawa-Haas et al. 2016).

 Cooperation will be defined here as ‘all interactions or series of interactions that, as a rule (or “on average”), result in net gain for all participants’ (Noë 2006). These interactions are widespread in nature and an essential ingredient of, for example, cooperative hunting (Bailey et al. 2013; Boesch 1994; Bshary et al. 2006; McMahon and Evans 1992) and cooperative breeding (Solomon and French 1997; Koenig and Dickinson 2004; Gilchrist 2007). Cooperative behaviour has also been found and studied in interspecific interactions among vertebrates, for example cleaning mutualisms (Bshary 2001), cooperative hunting by mongooses and hornbills (Rasa 1983) or groupers and moray eels (Bshary et al. 2006), and anti-predatory associations formed by mixed-species groups of red colobus (*Procolobus badius*) and Diana monkeys (*Cercopithecus diana*) (Noë and Bshary 1997).

A factor commonly found to affect cooperation success in animals has been tolerance, defined here as the acceptance by a dominant individual of a subordinate’s use of a resource controlled by the dominant, e.g. a food patch. Dominant individuals that show low or no tolerance towards sharing the reward will eventually cause the subordinate to defect, ceasing cooperation (Engelmann et al. 2015; Massen et al. 2015). In cooperative string-pulling tasks, tolerance was positively correlated with success in experiments with chimpanzees, *Pan troglodytes* (Melis et al. 2006b), Barbary macaques, *Macaca sylvanus* (Molesti and Majolo 2015a), marmosets, *Callithrix jacchus* (Werdenich and Huber 2002), rooks, *Corvus frugilegus* (Seed et al. 2008), and ravens, *Corvus corax* (Massen et al. 2015). Additionally, other social parameters related to the dominant–subordinate relationship have been found to be factors in cooperation. Spotted hyenas, *Crocuta crocuta*, cooperated better when the dominant showed less aggressive behaviour (Drea and Carter 2009), while ravens cooperated better when the dominance rank difference between the partners was higher (Massen et al. 2015).

However, attributes like tolerance and dominance are not mutually exclusive and are often very difficult to disentangle. Dominance is usually established on the basis of agonistic interactions (Drea and Carter 2009; Scheid and Noë 2010), while tolerance is often quantified by the distance between a dominant and a subordinate in a shared reward task: decreasing distance shows increasing tolerance of the dominant towards the subordinate (Hare et al. 2007; Massen et al. 2015). The distance, however, might also be influenced by food-sharing tendencies which have been shown to correlate with behaviours used to measure affiliation (King et al. 2011; Eppley et al. 2013). In the current study, we attempted to simplify the situation by using a slightly different apparatus to those in previous loose-string studies (see ‘Methods’ for details).

Another aspect that may predict the success of a future cooperation, notably in the following trial of the same experimental session, is the division of the resource produced from the successful cooperation in the previous trial. In many studies using cooperation tasks, the subjects’ willingness to continue cooperation was directly dependent on being rewarded (Mendres and de Waal 2000; Melis et al. 2006b; Seed et al. 2008). Moreover, in chimpanzees and ravens, subjects were more likely to defect in a cooperation task when the previous reward division with the same partner was unequal and not in their favour (Engelmann et al. 2015; Massen et al. 2015). Therefore, in this study reward division (absolute number of rewards per subject) and reward equity (number of rewards relative to that of the partner) were both analysed as possible factors influencing the continuation of successful cooperative behaviour between subjects.

Here we present a string-pulling cooperation task undertaken with the kea parrot (*Nestor notabilis*), which is only distantly related to the corvids and true parrots, such as the African grey (Wright et al. 2008; Jarvis et al. 2014), and has hitherto not been tested in a loose-string task. Similar to corvids and African grey parrots, kea are relatively large-brained and their brains pack an equal or greater number of neurons than primates (Olkowicz et al. 2016). Moreover, they have been shown to solve tasks requiring both sophisticated motor and reasoning skills (Huber and Gajdon 2006; O’Hara et al. 2012). Their natural social structure and behaviours allow for a gregarious nature (Diamond and Bond 1991), while they still form strong affiliations between specific individuals, resulting in a social organization with fission–fusion dynamics comparable to chimpanzees and spider monkeys (Jackson 1960; Symington 1990; Diamond and Bond 1999; Aureli et al. 2008).

We investigated what predicts success in a cooperation task between kea in dyads using the loose-string paradigm. We physically separated the birds to exclude directional social effects, i.e. tolerance and aggression were no longer possible. However, dominance is a lasting feature of a relationship, which can be instrumental in modifying the subordinate’s behaviour in consecutive sessions and has been shown to affect cooperation in a bird species (Massen et al. 2015). Therefore, despite aggressive dominant behaviour not being possible during the trials, dominance ranking remained a factor in our analysis. During training, a control was implemented to examine whether the birds showed understanding of both the mechanism of the task and the need of the partner. Moreover, the experiment was designed in such a way that the birds cooperated for a sharable reward, allowing us to analyse the effects of reward division in trial *n* − 1 on the likelihood of them cooperating again in trial *n*. Our predictions were as follows: (1) pairs with stronger affiliative relationships would succeed more often in the cooperative task; (2) a more balanced reward division would be associated with a greater likelihood of (subsequent) cooperation.

## Materials and methods

### Ethical note

The experiment was approved by the University of Veterinary Medicine Vienna’s institutional ethics committee (17/02/97/2012) in accordance with Good Scientific Practice guidelines and national legislations. All subjects that participated in our experiments were housed in accordance with the Austrian Federal Act on the Protection of Animals (Animal Protection Act-TSchG, BGBl. I Nr.118/2004). Furthermore, as the present study was strictly noninvasive and based on behavioural observations, none of the experiments were classified as animal experiments under the Austrian Animal Experiments Act (×2, Federal Law Gazette No. 501/1989) and consequently did not require further permission.

### Subjects and aviary

Fourteen captive kea (*Nestor notabilis*), a parrot species endemic to New Zealand, took part in the study. In the wild, they congregate at spots of interest (e.g. locally abundant food source) forming large groups of up to 30 birds, comprising smaller family units and bachelor groups (Diamond and Bond 1999). These fission–fusion groups change frequently in composition and lack a linear hierarchy. Feeding in close proximity occurs with high frequency, while aggression is infrequently encountered, even at high-value food sources (Schwing 2010). Kea are highly neophilic and exploratory, especially in a group, but they are not known to cooperate in the wild (Jackson 1960; Diamond and Bond 1999).

Subjects were group-housed in an outdoor aviary (52 × 10 × 6 m) at the Haidlhof Research Station, near Bad Vöslau, Austria. All subjects had been in the group since hatching. All of the birds had been involved in other behavioural experiments; however, none of our subjects took part in a previously described cooperation experiment (Tebbich et al. 1996). Supplementary material provides detailed information on the birds’ participation in the different parts of the experiment and more background information about the birds (age, sex, rank).

The experiments were conducted from January to May 2014 in an experimental compartment (6 × 10 × 6 m), which was visually isolated from the rest of the aviary by sliding opaque walls. Aside from the experimental apparatus (described below), this compartment was equipped with the same interior furnishings as the rest of the aviary and was fully accessible outside of testing times, allowing the kea to retreat between experimental sessions to familiar higher perches. The kea were fed three times a day with a mixture of seeds, fruits and vegetables, and a protein source (eggs, meat, or cream cheese depending on the season) once a day. Water was provided ad libitum, also during testing.

### Apparatus

**Fig. 1:**
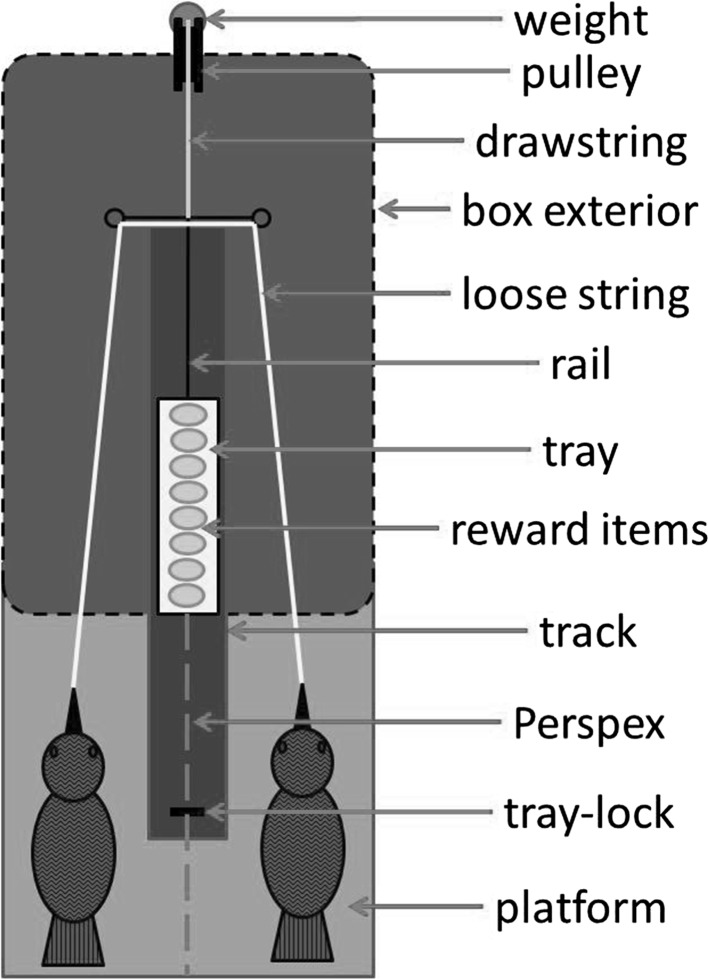
*Top-view* diagram of the cooperation box. Two kea separated by Perspex were required to pull on the string ends simultaneously until the tray moved forward enough to lock in place; only then could the subjects let go and divide up the reward

The apparatus, which was based on the design of Scheid and Noë (2010), consisted of a wooden box (80 × 150 × 100 cm) containing a metal track with a sliding tray on which the rewards were placed (Fig. 1). Eight small commercially available parrot food pellets (Nutribird G14 Original, Versele-Laga, Deinze, Belgium) stuck to the tray with cream cheese were used as rewards for each trial. These pellets were chosen as they are of uniform size and colour, and preferred over high-value food items from the birds’ normal diet (Schwing et al. in prep.). A transparent Plexiglas window between the two sides allowed the birds to see each other, but prevented any physical contact between them. Note, however, that a cut-out in this Plexiglas window did allow both birds to access to the metal plate with the sliding tray on which the rewards were placed. The string ends were placed on the box’s test platforms. The basic loose-string principle (Hirata 2003) applied: if the string was pulled from only one side, it would slide out without moving the tray, whereas if both ends were pulled simultaneously, the tray would slide. Both subjects had to continue pulling until the tray locked in place at a maximal emergence point (Fig. 1); if either bird let go of the string before the tray had reached this point, an attached weight (200 g) slid the tray back out of reach, often pulling the released end of the string out of reach. Once the tray was locked in place, the rewards on top of it were reachable by both subjects, giving them an equal chance to obtain each of the reward items. Transparent Plexiglas plates, secured with a small hook, blocked the access to the string between trials. A trial started as soon as these plates were raised. The plates were spring-loaded and released by pulling on a long cord, allowing simultaneous release of both plates and at least 2 m distance between the experimenter and the birds.

### Procedure

#### Individual training and control trials

Before starting with the dyadic tests, subjects (*N* = 14) underwent individual training in order to get used to the box and the loose-string system. Individual training consisted of a total of eight sessions, at four consecutive trials per session, with three different situations: in two trials the experimenter held the string and pulled simultaneously with the bird (cooperation training situation: cooperation), in one trial the experimenter was 1.30 m away from the box and did not pull the string (position control situation: no cooperation), and in one trial the experimenter was near the box with her back turned towards the platform and did not pull the string (orientation control situation: no cooperation); the order of the four trials was randomized. A trial ended in one of four ways: (1) the subject manipulated the string, locking the tray and reaching the reward (cooperation training); (2) the subject manipulated the string, pulling it out (position and orientation control); (3) the subject landed on the test platform but failed to manipulate the string (all situations); (4) 5 min after the start of the trial (if the bird did not climb onto the test platform) (all situations). The two controls allowed us to examine in more detail what aspect of the human partner’s behaviour the kea paid attention to in this cooperation task training. Success in control 1 but not 2 would have suggested the kea paid attention to the orientation of the human partner, but not the position from where they could cooperate. Success in control 2 but not 1 would have suggested the kea paid attention to the position of the partner, but not the orientation required to participate.

#### Dyadic tests

Ten individuals took part in the dyadic test sessions; the four other birds initially participated too, but lost motivation due to breeding activities. We aimed to test all 45 possible dyadic pairings twice to balance the position of the birds (right or left side of the box). Unfortunately, we were unable to test one dyad at all and one dyad could be tested only once, because of breeding activities and because one individual refused to participate any further. Each test session for each dyad consisted of eight trials. A trial started as soon as the transparent Plexiglas plates were raised, allowing access to the string ends. The end of a trial was determined by the same criteria as the training session trials. The experimenter was present during trials, but stood at a distance of approximately 2 m behind the apparatus to prevent inadvertent cueing or other ‘Clever Hans’ effects.

#### Behavioural and background data

For the training, we coded whether the birds successfully cooperated with the human partner, and did or did not pull when the human partner was not holding the other end of the string (orientation and position controls). For the dyadic test, we recorded whether individuals went onto the platform and both held the string at the same time but did not lock the tray properly (‘cooperation attempt’), locked the tray successfully (‘success’), and, if successful, how they divided the reward. They could share the eight small pellets per trial in any of nine different ways; this gave us the reward division (RD) for each bird, i.e. the proportion of rewards eaten (ranging between 0 = subject did not get to take a reward and 1 = subject took all rewards). We then calculated the reward equity (RE) between two birds for each trial:

$${\text{RE}} = 1{-}2 \times \left| {0.5{-}{\text{RD}}} \right|$$

Reward equity ranges between 1 = total equity (4–4 division) and 0 = total inequity (0–8 and 8–0 divisions) and is the same for both birds; here we tried to make the distinction between a parameter that can measure differences for a specific bird (reward division) and the absolute inequity of each trial (reward equity), to examine in more detail the effect of the rewards on future cooperation. Note that, however, reward division was not visible in every trial, and hence, reward equity could also not always be calculated either.

We gathered observational data using continuous focal animal sampling (Altmann 1974), to assess affiliative and dominance relationships, as these parameters of relationship quality might have played a role in cooperation success despite the lack of directional social behaviours. Focal protocols were performed on a weekly basis, with each bird followed for 2-min continuous sampling including three instantaneous scans at 1-min intervals. The data used covered the time from May 2013 until June 2014 (55 samples per bird). From these protocols, we extracted two parameters: the nearest-neighbour values to calculate an affiliative score, and the number and direction of displacements for each pairing in order to calculate the rank of the birds during the experiment.

Each displacement of one bird by another during the focal protocols provided two data points, one for each bird. We calculated the Clutton-Brock Index (CBI) from these, since this has previously been used in the determination of the hierarchy in wild kea (Gajdon et al. 2006). For an individual *i*, we used the formula:

$${\text{CBI}}_{i} = (B + b + 1) / (A + a + 1)$$

where *B* is the number of individuals *i* displaced; *b* is the number of individuals displaced by birds subordinate to *i*; *A* is the number of individuals that displaced *i*; and *a* is the number of individuals displacing birds dominant to *i*.

The identities of nearest neighbours, defined as any individuals within one metre of the focal bird during protocol scans, were extracted from the focal samples for all subjects. The absolute number of protocols during which two individuals were recorded as nearest neighbours was used as the affiliative score in the analysis.

## Data collection and analysis

Each trial was recorded with a digital video camera (Légria HFR 37, Canon, Fujio Mitarai, Japan). We used Solomon Coder^®^ v.12.09.04 software (© 2013 by András Péter) to code the behaviours on the videos. Coding reliability was tested by comparing the experimenter’s (EJ) scores of reward division with those of two naïve observers who recoded 15.9 % of the original videos. We found a high level of correlation between the scores obtained by the two naïve observers and the experimenter (Spearman’s correlation, for the first naïve observer: *ρ* = 0.86, *N* = 60, *p* < 0.001 and for the second naïve observer: *ρ* = 0.81, *N* = 62, *p* < 0.001).

To assess what affected the number of successful cooperation trials per dyad and the number of cooperation attempts per dyad, we built generalized linear mixed models (GLMMs). These response variables were tested against fixed parameters: sex of the subject, sex combination of subject and partner, rank of the subject, rank difference between subject and partner, kinship between subject and partner (relatedness, *r* > 0.25 = kin; Chapais 2001), affiliation score of subject and partner, age of subject, and session number. In addition, we ran a binomial GLMM with a logit link function to test how the reward equity of a previous trial influenced the chance of cooperation being successful in the next trial. Finally, to assess factors influencing reward distribution equity we ran a GLMM, testing RE as the response variable against sex of the subject, sex combination of subject and partner, rank of the subject, rank difference between subject and partner, kinship between subject and partner, affiliation score of subject and partner, and age of subject. As we dealt with repeated data, we structured all our data to be nested in each individual, which in turn was nested in its partner for a specific dyad. Consequently, we entered subject identity and partner identity as random variables into our models.

We ran models including all main effects and several reduced models and selected the best-fitting model by comparison with the corrected Akaike information criteria (cAIC). For the sake of clarity, here we only report the best-fitting models.

Statistical tests were carried out with R (version 3.0.2, R Development Core Team, University of Auckland, New Zealand) and SPSS (version 21.0, IBM, Armonk, USA) statistical software. All reported *p* values are two-tailed, and the significance threshold was fixed at *α* ≤ 0.05.

## Results

During the individual training, we observed 97 % success in cooperation trials (217 out of 224, cooperation training) and only 4.5 % success in control trials (3 out of 112, position control and 7 out of 112, orientation control; successful = subjects did not pull) among the fourteen participants.

In the dyadic test set-up, we found that despite each subject being successful in some trials and 61.4 % of the pairs (27 out of the 44 pairs) successfully cooperating at least once, only 18.9 % (132 trials out of 696) of all trials were successful.

**Fig. 2:**
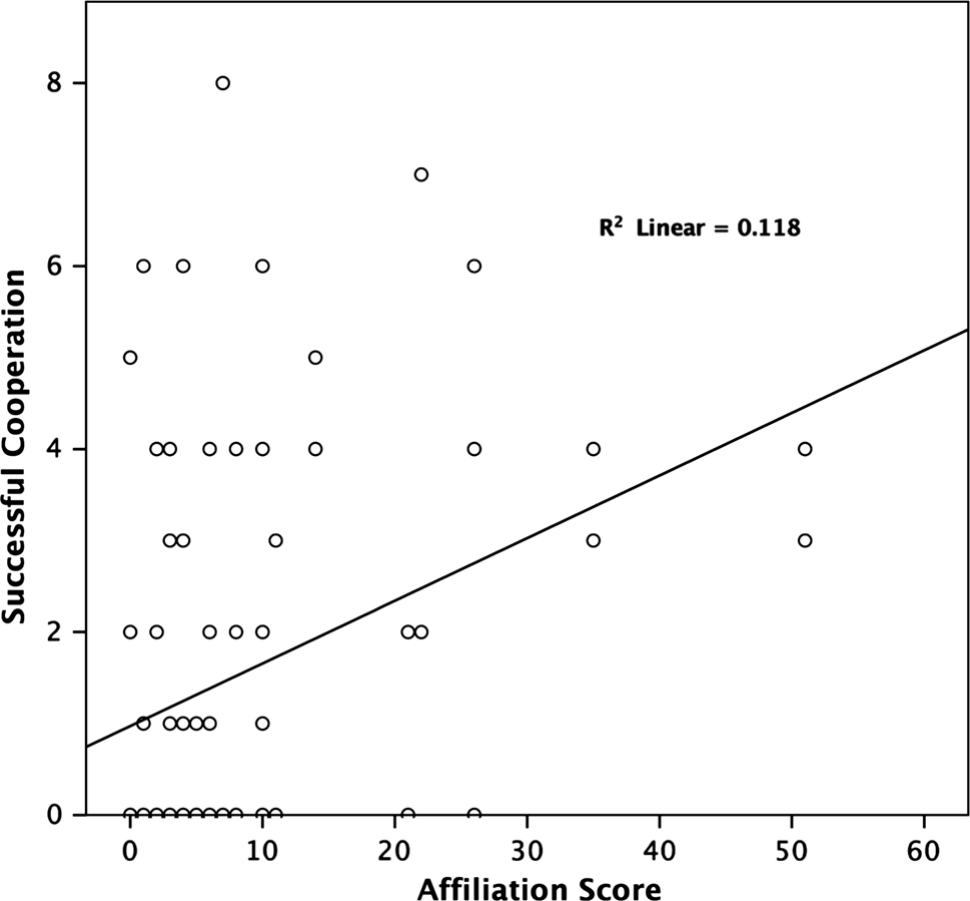
Relation between successful cooperation and affiliation score. The higher the affiliation score between two partners, the more successes that dyad had in the task

**Fig. 3:**
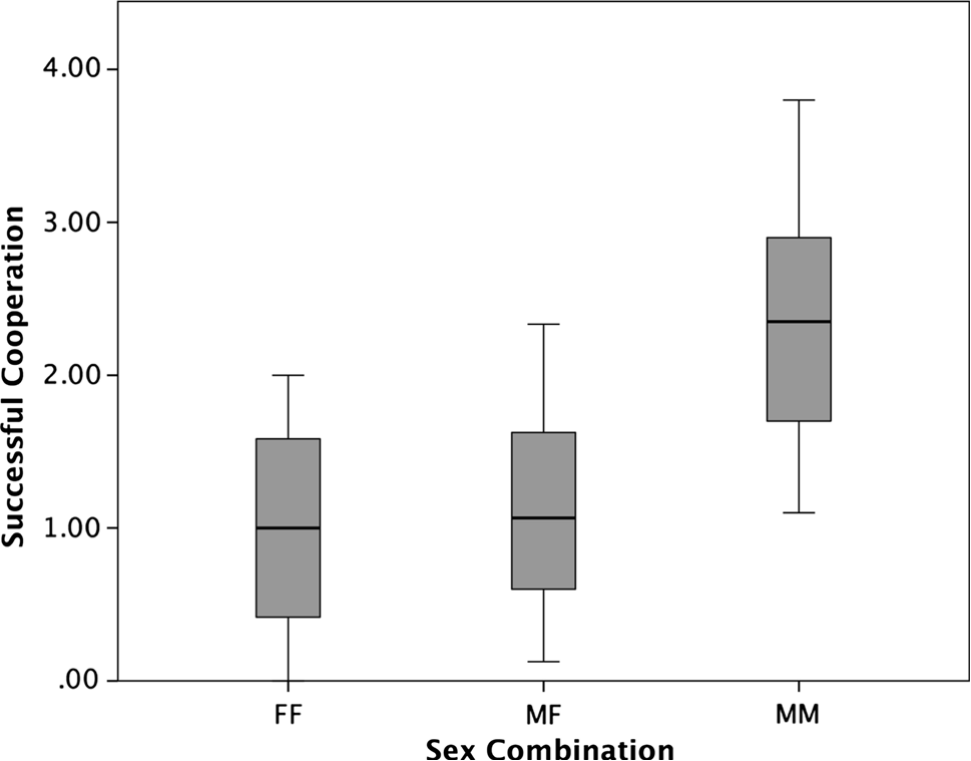
Median, interquartile range, and range of successful cooperation trials of female–female dyads (FF), male–female dyads (MF), and male–male dyads (MM). Dyads with two male partners seemed to cooperate more often than those with one male and one female or both female partners

**Fig. 4:**
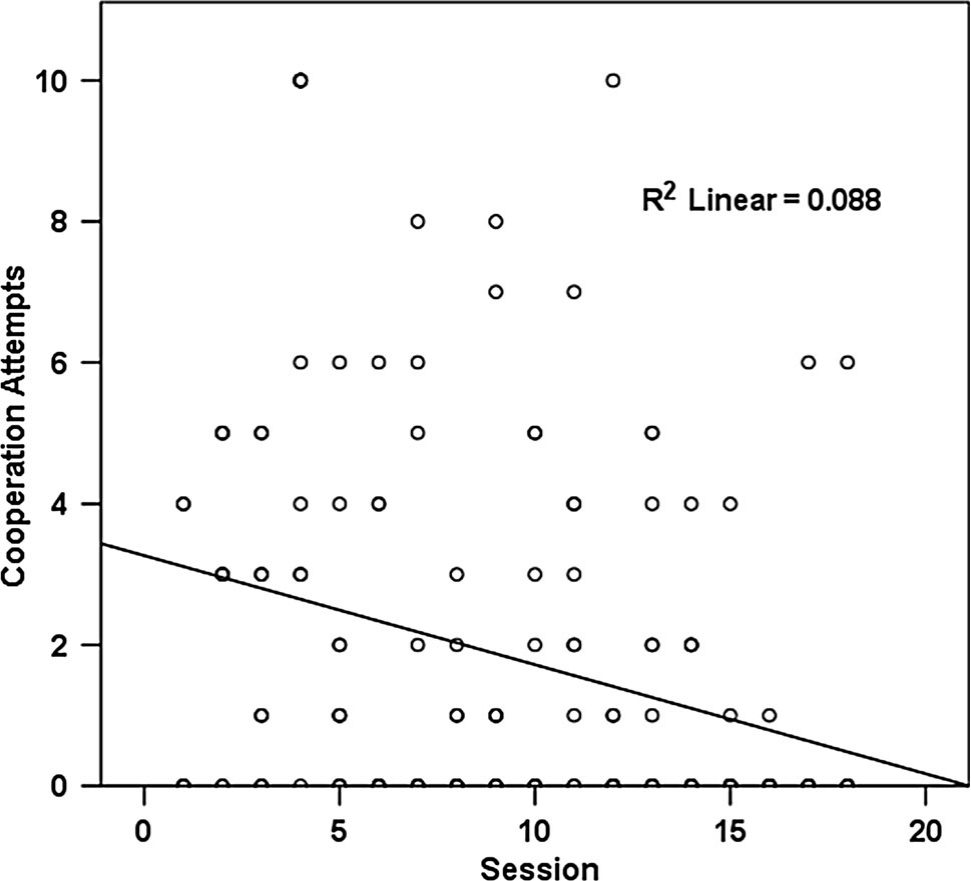
Relation between cooperation attempts and session. The kea dyad attempts to cooperate decreased with increasing session number

The best-fitting model on overall cooperation success showed two significant main effects and one near significant trend. The affiliation score of a dyadic pairing had a significant positive effect on the overall success of their cooperation (GLMM, *β* ± SE = 0.034 ± 0.014, *F*_1,168_ = 6.165, *p* = 0.014; Fig. 2); i.e. the higher the affiliation score of a dyad the more successful they were in the cooperation task. We found a significantly negative effect of session number on cooperation success (GLMM, *β* ± SE = −0.109 ± 0.025, *F*_1,168_ = 18.253, *p* < 0.001). Additionally, there was an effect of sex combination on cooperation success, albeit nonsignificantly (GLMM, *β* ± SE = 1.170 ± 0.945, *F*_2,168_ = 2.761, *p* = 0.066), suggesting that males cooperate better with other males than with females, and also better than females among each other (see Fig. 3). The negative effect of session number on cooperative success may have been due to a reduction in general motivation over time, and therefore, we investigated whether the number of attempts also diminished in the course of the experiment. The best-fitting model on cooperation attempts indeed corroborated that hypothesis, since we found a significant negative relationship between session number and number of attempts (GLMM, *β* ± SE = −0.146 ± 0.033, *F*_1,167_ = 19.295, *p* < 0.001; Fig. 4). In addition, this model confirmed the positive effect of affiliation score, albeit as a nonsignificant trend only (GLMM, *β* ± SE = −0.034 ± 0.018, *F*_1,167_ = 3.638, *p* = 0.058).

**Fig. 5:**
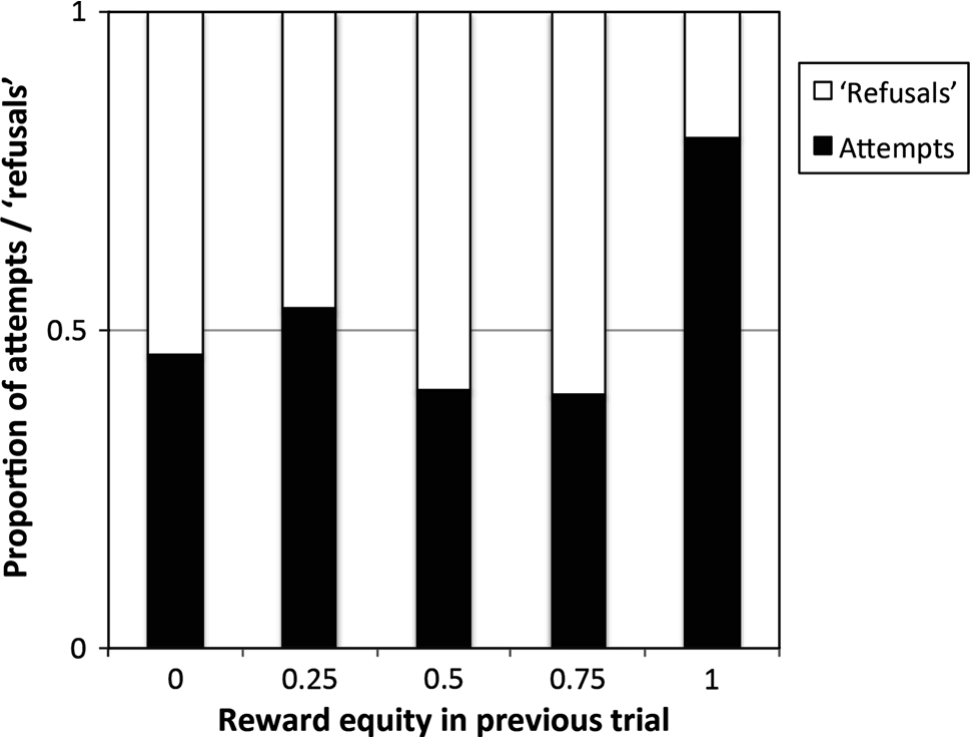
Proportion of attempts—(*black part of the bar*) and ‘refusals’—(*white part of the bar*) to cooperate after reward equity in the previous successful ranging from 0 (total inequity) to 1 (total equity). Dyads which shared equitably were more likely to attempt to cooperate in the subsequent trial

A binomial analysis on cooperation success per trial showed no significant effect of the reward equity of the previous trial (GLMM, *β* ± SE = −0.013 ± 0.009, *F*_1,384_ = 1.901, *p* = 0.169). There was nevertheless a trend, albeit nonsignificant, that the reward equity of the previous trial affected the likelihood of an *attempt* to cooperate in the next trial (GLMM, *β* ± SE = −0.017 ± 0.010, *F*_1,384_ = 3.188, *p* = 0.075; Fig. 5); i.e. individuals were more likely to attempt to cooperate when the rewards in the previous successful trial were divided equally compared with when they were divided unequally. Note that, although included in the full model, reward division did not contribute to the best-fitting model.

Finally, regarding what affected reward equity, the best-fitting model was the null-model; i.e. none of our parameters seemed to predict reward equity.

## Discussion

We showed that kea spontaneously solved the cooperative loose-string paradigm when paired with a human, and thereafter could also do this with conspecifics. When paired with conspecifics, the kea attempted to cooperate more with affiliates and were also more successful doing so with affiliates than with nonaffiliates. However, they did not seem to understand either the mechanics of the loose-string apparatus or the need of a partner, as they failed both types of control in the training. This suggests that they paid little attention to the presence or actions of the partner. Nevertheless, the kea were not completely inattentive, as we showed a trend that individuals were more likely to attempt to cooperate again after a successful cooperation trial in which the rewards were divided equally than after a successful trial in which the rewards were divided unequally. This could be more parsimoniously explained, however, as a reinforcement of both individuals independently by a satisfactory amount of rewards in the previous trial. Furthermore, we found a trend that dyadic pairings with only male partners had higher success rates. We also found that the keas’ motivation to perform the task decreased over time.

In previous experiments, tolerance has been a requirement for many species to cooperate successfully (Petit et al. 1992; Melis et al. 2006b; Hare et al. 2007; Seed et al. 2008; Drea and Carter 2009; Péron et al. 2011; Suchak et al. 2014; Massen et al. 2015; Molesti and Majolo 2015b), whereas in this study tolerance was excluded as a factor. In the absence of the need for tolerance by the dominant member of a pair, affiliation in turn influenced cooperation success. This contrasts, however, with recent results on ravens, which showed that in the absence of the need for tolerance by the dominant, due to a physical separation between the two cooperation patterns, relationship quality (cf. affiliation) did not influence cooperation success (Asakawa-Haas et al. 2016). This might stem from a greater understanding of the task by the ravens in Asakawa-Haas et al. study, which sometimes waited for a partner to cooperate. This suggests that without an understanding of the mechanism or need for a partner, the factors influencing the kea in this cooperation task are the same as would influence a food-sharing situation.

Furthermore, reward equity had a positive effect on the likelihood of cooperation attempts, albeit nonsignificantly. However, none of our measures predicted what caused rewards to be divided more or less equally. Therefore, additional research into what affects reward division is needed, as reward division effects on cooperation are rarely tested. So far, only three studies of capuchin monkeys (De Waal and Davis 2003), chimpanzees (Engelmann et al. 2015), and ravens (Massen et al. 2015) have shown that individuals are more likely to cooperate if the reward division in the previous trial with that partner was equal rather than unequal. However, rewards in these studies were either clumped or there were only two rewards, making reward division binomial; i.e. either it was divided equally or one individual took everything and the other got nothing. In contrast, we provided eight dispersed rewards that could be divided in multiple ways, and show that this variable predicts (albeit only as a trend) whether two individuals will attempt to cooperate; i.e. when equality increases, so does the likelihood of a cooperation attempt in a subsequent trial.

In contrast to earlier experiments (Drea and Carter 2009; Scheid and Noë 2010; Massen et al. 2015), we found no effects of dominance or rank. There are two major differences in our set-up compared with previous experiments that may account for this discrepancy. First, the subjects were physically separated (by wire mesh and a plastic window), so the dominant bird could not physically affect the subordinate during each session. Second, the reward was dispersed, as opposed to clumped. Spreading out the reward led to lower frequencies of conflict, less opportunities for monopolization by more dominant animals, and higher frequencies of successful cooperation in studies with primates (De Waal and Davis 2003; Melis et al. 2006b). We did find a trend for males to cooperate more with each other than with females or than females with each other. This could stem from a higher similarity of actions performed in males, i.e. both grabbed their string end as quickly as possible. In a dyadic set-up requiring coordinated action, this would affect the outcome the strongest in a male–male pairing. Adult males are also bolder and more exploratory than females and are the sole provider of food for the female and chicks during nesting (Diamond and Bond 1999). This could have selected for a heightened sensitivity to local and stimulus enhancement effects by the actions of other males, as they could learn from conspecifics about new or restricted food sources.

In a string-pulling task, we can deduce that the subject understands the role of the partner if it refrains from pulling when the partner is absent. Waiting for the partner to pick up the string before pulling is a clearer demonstration of understanding of the mechanism. The kea did neither in any phase of this study. This explains the low success rate both in the control trials of the individual training (4.5 %) and in the dyadic pairing tests (18.9 %). The low success rate, in turn, explains the significant decrease in attempts to cooperate over the course of the experiment, as motivation dropped probably due to lack of reward (cf. De Waal and Davis 2003) in the unsuccessful trials. The latter contrasts with findings in ravens, whose motivation to perform the task increased over time (Massen et al. 2015). This may reflect the different attitude towards novel items of kea (neophilic) and ravens (neophobic).

Like all other bird species tested so far (Seed et al. 2008; Scheid and Noë 2010; Péron et al. 2011; Massen et al. 2015), but unlike, for example, chimpanzees (Melis et al. 2006b), the kea thus did not spontaneously show understanding of either the role of the partner or the mechanism behind this cooperation task. Seed and colleagues (Seed et al. 2008) suggested that chimpanzees are more successful at cooperative string-pulling than the rooks they tested, because the chimpanzees have more a complex social structure. However, this argument has become less likely with cumulative results from other birds. Kea, rooks, and ravens all live in large groups, with ever-changing compositions, that can further subdivide during foraging and then remerge to larger flocks suggesting complex fission–fusion dynamics (Jackson 1960; Diamond and Bond 1999; Aureli et al. 2008; Braun et al. 2012; Jolles et al. 2013). This suggests that all three species have highly complex social structures, with both competitive and cooperative relationships. Despite this, the kea, and the ravens in a recent study (Massen et al. 2015), showed no greater understanding of the task or partner’s role than rooks did (Seed et al. 2008). It should be noted though that comparisons about understanding should rely on equal training and experience with the paradigm and similar ways of testing this understanding, something that has not been the case in the studies that are currently available. For example, the chimpanzees in the seminal study of Melis and colleagues (2006b) received extensive training on (a) how to use the apparatus by themselves with the ends of the ropes in reach and (b) waiting for a partner after increasingly long delays. Although the rooks (Seed et al. 2008) did receive individual training on how to use the apparatus, the ravens (Massen et al. 2015) and kea (this study) did not. Notably, none of the bird species got any training with regard to the delayed arrival of a partner, and the rooks and ravens failed delay tests (Seed et al. 2008; Massen et al. 2015), though it seems that with more exposure to the task, the ravens learned to wait (Asakawa-Haas et al. 2016). A caveat in our study is that we did not test for the kea’s ability to wait for a partner. Our controls both involved completely unresponsive humans, and there was no opportunity for the kea to procure a reward in these controls. Consequently, the kea needed to inhibit pulling for 5 min in these control trials, which might have been very difficult, even if they had understood the task. Finally, the complexity of our apparatus, i.e. the limited visibility for the kea to follow the trajectory of the string (see Fig. 1), might have contributed to their inability to understand the mechanism presented. These results suggest that the cooperation task, without an understanding of the mechanism or need for a partner, acts as a complex food-sharing situation.

Although we are no closer to explaining the lack of understanding of the mechanism within the avian clade, this research clearly showed that affiliation is an important factor governing cooperation, as it allows for success in the absence of understanding and/or physical contact. Nevertheless, the effects we have found of affiliation and reward division on the initiation and maintenance of cooperation require further investigation.

## Electronic supplementary material

Below is the link to the electronic supplementary material.

Supplementary material 1 (XLSX 140 kb)
